# Learning echocardiography- what are the challenges and what may favour learning? A qualitative study

**DOI:** 10.1186/s12909-019-1656-1

**Published:** 2019-06-13

**Authors:** Anna Dieden, Elisabeth Carlson, Petri Gudmundsson

**Affiliations:** 1 Department of Biomedical Science, Malmö University, Malmö, Sweden; 2 Department of Care Science, Malmö University, Malmö, Sweden

**Keywords:** Echocardiography, Feedback, Pedagogy, Teaching, Theory-practice-gap, vLAB

## Abstract

**Abstract:**

**Background:**

Echocardiography is a frequently used imaging modality requiring extensive training to master. In order to develop curriculums and teaching material fully favouring students learning within echocardiography, this study aims to investigate students’ experiences of learning echocardiography, focusing on that which is perceived as the main challenges as well as what might aid learning within the area. The findings could serve as a foundation in the development of new teaching material or curriculums.

**Methods:**

A qualitative study was performed with data gathered through two audio-recorded focus group interviews with four third year students from the biomedical laboratory programme at Malmö University in each group. Data was analysed by manifest content analysis.

**Results:**

Findings were clustered into two categories reflecting the main findings in the text – practical skills and bridging the theory-practice-gap. Students expressed that main challenges when initially learning echocardiography were the projections and handling the probe as well as connecting ultrasound physics and measurements to practical application. Things that aided their learning were immediate feedback, “playing” with the ultrasound machine, video lectures, the possibility to swiftly alternate between practice and theory as well as the learning by their mistakes in a risk-free environment.

**Conclusions:**

This study shows the main challenges when initially learning echocardiography and what might be helpful during the learning process. These findings may be useful when developing curriculums or new teaching material within echocardiography. One suggestion might be to develop digital resources such as virtual laboratories (vLABs).

## Background

Cardiovascular disorders are today by far the leading global cause of death [1]. As the prevalence of these diseases increases, so does the need for diagnosis. Echocardiography is the most commonly used imaging modality within clinical cardiology, as the information it provides is extensive and immediate as well as cost-effective, non-invasive and accessible. The disadvantage is that it is user-dependent and requires extensive practice to master [2–4]. The development of medical ultrasound, since its origin 70 years ago, has been extensive. Modalities such as M-Mode, Doppler, 2D as well as 3D are now an integral part of a routine examination [5, 6]. The ultrasound practitioner needs an understanding of the physics behind these modalities, as well as adequate knowledge of the anatomy, physiology and pathophysiology of the heart. In addition, satisfactory technical competence and practical skill are required in order to perform an examination that is both useful and clinically relevant [3]. Echocardiography education worldwide is very different depending on the profession subject to the learning process and on the purpose [2, 4, 7]. However, the initial challenges of handling the transducer and connecting it to the anatomy of the heart, as well as understanding the basics of ultrasound physics are the same. It can be challenging to obtain and assess information from a two-dimensional image in a three-dimensional organ. During training for basic level, when the practitioner is expected to be able to perform a full routine examination followed by writing a report, the European Association of Echocardiography recommends a minimum of six months of active full-time training as well as performance of a minimum of 350 transthoracic echocardiographic examinations [4], putting into perspective how demanding the examination can be to master.

To make use of the time spent training and to design a curriculum fully favouring the students’ learning, it would be beneficial to explore what the students perceive as challenging in the echocardiography learning process as well as what they feel is helpful. In addition, such results could serve as a foundation in the development of new teaching material, such as a virtual laboratory (vLAB) within echocardiography. Previous studies on vLABs [8, 9] have shown to increase students’ knowledge, intrinsic motivation and self-efficacy and could therefore serve as a meaningful pedagogical tool when learning a knowingly complex examination like echocardiography. Studying the students’ experiences in learning echocardiography in order to use it to develop curriculums or vLABs would be a way of inviting them as partners in the development process. Several studies have found numerous positive effects of using students as partners, such as increased student engagement, motivation and ownership of learning as well as the development of newer or better teaching or curriculum material [10]. Even when developing new medical equipment, it is not unusual to take the users’ perspectives into consideration. According to Shah and Robinson [11] users (in this case, students) can provide meaningful input in different stages of the development of new medical equipment.

There are several studies showing the benefits of using simulations, mannequins or e-learning within echocardiography education [12–16]. These studies often highlight and comment on the difficulties of learning echocardiography. However, they do not show or study *why* or *what* the learning difficulties might be. Therefore, the purpose of this study is to gain greater understanding of the experience behind learning echocardiography, focusing on the challenges as well as what may favour learning.

## Method

A qualitative research design, utilising focus group interviews as data collection method, was employed as a means to explore the students’ subjective experience of learning echocardiography. Focus group interview was chosen due to its ability to instantly provide information on similarities and differences in perspectives between participants who share experiences, compared to individual interviews where such conclusions must be drawn after the interviews by analysing separate statements from the participants [17].

### Study population and data collection

The study was performed with students at the biomedical laboratory programme at Malmö University. The curriculum consists of a three-year bachelor programme, the aim of which is to form a foundation to independently be able to plan, implement and interpret analyses and examinations. For students studying clinical physiology in the final year, echocardiography is one of these examinations. At the end of the course the students are expected to be able to perform a basic ultrasound examination including rudimental assessment of its information. In order to be able to do this they need to be able to explain ultrasound physics and describe ultrasound methodology. The curriculum is based on the principle of constructive alignment [18] and the teaching is both theoretical and practical. The practical teaching is performed by teachers with several years of clinical experience within echocardiography. The students are given an introduction, beside the ultrasound machine, on how to work the machine and how to handle the transducer in order to obtain the various projections. They are also given a document that specifies the projections and methods to be used in a standardised examination. The students are then given a certain amount of training time at the machine, with a teacher available when questions and difficulties arise. The presence of teachers is greater at the beginning, when introducing the method and they are thereafter available when called on. In addition, students have free access to a training laboratory where they can practise without the teacher supervision, to allow increased student independence. The practical part is assessed by a practical examination where the students need to show that they understand how to handle the machine and the transducer, as well as which projections and methods are relevant when wanting to obtain certain information. The theoretical part is assessed by a computer-based exam in which they are given a case (including echocardiography loops in motion) where they are to assess the diagnostic information as well as answer questions concerning the related ultrasound physics.

We used convenience sampling inviting all year three students enrolled in the clinical physiology course in October 2017 (*n* = 15). Out of these students, eight voluntarily accepted to participate in the study. None of the students had previous experience of ultrasound prior to the course.

The study was performed after the course had been completed and the students had been informed of their grades. They were divided into two groups (4 + 4). The author who also was one of the course teachers moderated both interviews and an observer sat in on the interview. The observer, a teacher who had not participated in the course, did not participate actively in the interview but took notes on what was discussed during the interviews. The students were encouraged to share experiences, thoughts and memories connected to their learning of echocardiography. The interviews started with one open or broad question “To start off I would like every one of you to briefly share your experiences of learning echocardiography” which started a discussion among the students. Supporting questions such as “What did you mean by…” or “Could you please explain a bit more…” were then asked only when the discussion was led into areas needing clarification. In order to further stimulate the discussion, remove focus from the moderator and instead direct focus onto the topic various stimulus material related to the topic discussed can be helpful [19]. In this study the handouts from the teaching were available during the interviews, key echocardiographic words were written on the boards and an echocardiography apparatus was stationed in the room. Initially everyone was invited to briefly and very openly share their experiences from their learning. They were encouraged to let the discussion circulate around difficulties in learning echocardiography as well as what was helpful, or ideas of what could have helped their learning. The students were encouraged to write key words from their discussions on the board. The interviews took place at the university, lasted 64 and 74 min respectively and were performed using audio recording.

### Analysis

As a starting point for the analysis the interviews were transcribed by the first author, followed by independent reading by the three authors (AD, EC, PG). The text was reread several times to obtain a sense of the whole. Two of the authors were familiar with echocardiography, representing an internal perspective and one of the authors was not, representing an external perspective. Based on the transcribed text, the notes of the observer and what the students had written on the whiteboard, a manifest content analysis was conducted. The inductive approach was performed with a focus on the challenges of learning echocardiography as well as what favoured learning. Meaning units where identified and thereafter sorted and coded. The codes where then grouped into categories, reflecting the central message in the texts [20]. To ensure credibility the preliminary analysis was returned to the students for respondent validation.

### Ethical considerations

In accordance with the Declaration of Helsinki [21] and the Swedish Research Council’s requirements regarding information and consent, informed consent was obtained prior to the interviews. All participants were given oral and written information concerning the study purpose, assurance of confidentiality and the right to withdraw from the study at any time. The students were assured that participation in the study would not compromise their professional relationship to the teacher, nor affect their final grade. However, all students had passed the course and were informed of this prior to the interviews.

## Results

Our findings illustrate students’ experiences of learning echocardiography with a focus on challenges in learning and what was expressed as support as well as their ideas of what could have further aided their learning. The results are a compilation of thoughts and experiences expressed within the two focus groups and are illustrated by two categories – *practical skills* and *bridging the theory-practice gap*, presented below. Subcategories were identified within each category and findings are illustrated by quotes from the students (S1–8) from the two groups (F1–2) respectively. After taking part of the initial analysis and suggestions for development, all students considered it to be representative of what they had expressed during the interviews.

### Practical skills

Initially, the students thought it was difficult to learn the projections. From a theoretical point of view, the projections were hard to link and relate to where in the heart the ultrasound beam cut. From a practical point of view, it was hard to know where the transducer should be placed, angled and turned in order to obtain certain projections.

#### Immediate feedback from the teachers

The students wished for more immediate response at the beginning while learning the projections in order to know whether or not they were doing it correctly. A recurring suggestion was for the teachers to be present at the start of the students’ practical training. Another suggestion was for an independent assessor to be present, correcting the students and asking them questions as they were practising. One of the students mentioned it would have helped if the ultrasound machine had some kind of indicator, showing them whether or not the transducer was placed correctly.

*F1S3 “Yes, exactly. That’s what I thought too. The teachers could have been more present, just so they…. So they could say “this is what you should do, this is how it should be”. But apart from that I thought it was a good course. Actually. And that we had a lot of opportunity to practise as much as we wanted to.”*

*F2S2: “When one was wondering “Where am I? What chamber am I looking at? Where is the regurgitation?”... and then… I don’t know… it had… I think it would have been good if you at the same time were given an indication on... WHICH diseases can I find here, connected to THIS valve?”*

#### Immediate response from fellow students

The students highlighted the advantage of working together and helping one another at the ultrasound machine. They saw great advantages in being three at the machine at the same time – one operator, one patient and one standing at the side – asking questions and comparing the projections on the display with the projections in the literature. They appreciated the fact that there was someone continuously asking questions and encouraging them to reflect on what they were doing and why.

*F1S2: “Yes, exactly. And then I realised quite early on that you have to be at least three. In echocardiography, to get anything out of it. That there is one who asks the one performing the echo… when you see this projection, what do you want to measure? And then tells you…”*

#### Access to pedagogical equipment

Something that helped the students learn the projections was their textbook on echocardiography which contains clear drawings of the projections and where the ultrasound beam cuts the heart in order to obtain each projection. The anatomical dummy of the heart helped the students to make a connection between the anatomy of the heart and the placing of the probe and location of the beam, while they were practising by the machine.

*F1S4: And the echocardiography book too… I used it quite a lot, the orange book. And it was good that it was accessible during the lab too. So we could look in it and compare the projections we got with the pictures in the book.*

*F2S7: Yeah, it was really…it helped me too, just to take the heart like that… how the beam cuts… short axis, long axis… F2S6: Exactly, hold it, and LOOK at it like this… yeah…*

#### Practical experience

The students thought it was helpful that there was a lot of practical training and that the ultrasound machine was so accessible. A recurring word during the interviews was “play”; they felt it was helpful to “play” with the ultrasound machine. It was positive that nothing could go wrong, there was no button that was “off limits”. They also wanted the teacher to hold her hand over the student’s while the student held the transducer in order to get an even better feel for handling the transducer.

*F2S8: “Mm, and then while practising echo it was like… I wanted to have someone beside me to be sure if I was holding the probe correctly… But then… no, I have to do this by myself to get more experience and to get a feel for it. Sometimes I did it the wrong way…. I placed the transducer wrongly just in order to see – what projection will I obtain? What will I see? What positions? What will the heart look like? Like that. But… it was easier for me to “play”, like you said, to find, and to practise on YOURSELF and it was easier”.*

*F1S1: “Well, it was really good to have the training laboratory where we were able practice. Really good.”*

### Bridging the theory-practice gap

The students found it challenging to integrate knowledge from class into practice with the machine. As previously mentioned, they found it hard to both theoretically and practically learn the projections but they also found it challenging to understand how and when certain measurements and concepts should be used in clinical setting. Also, they found it challenging to link the theory of ultrasound physics to practical performance with the machine.

#### Alternating between theory and practice

Generally, the students would have liked to alternate between theory and practice. Some students wanted practice prior to theory in order to first know *how* and then to know *why,* while other students wanted more theory before practice in order to better understand what they were doing when holding the transducer and practicing.

*F1S2: “Yes, because… I myself felt that – when I actually placed the probe… well then, I… eh... got a direct understanding of WHAT I saw on the screen. Because on the Wednesday when I went through the cases I just felt like “Ok, the right chamber is there and the left chamber is there, ok, ok.” But when I actually placed the transducer myself it actually became logical, why the chambers were where they were...So… I couldn’t connect the placing of the transducer with the projections until I placed the transducer myself”.*

*F2S2: “Mm, one somehow wanted to connect the visual to the theory… at the same time…”*

*F2S1: And THAT really helped a lot – the times we could book the training laboratory and examine each other. For example, when we read something, we could also put it into practice at the same time. And it helped… when we worked together.*

The students thought it was hard to link the ultrasound physics to the ultrasound machine and to practical performance. There were suggestions about connecting the practical learning with theory by teaching physics while using the ultrasound machine. If the theoretical ultrasound teaching had been conducted with an ultrasound machine from time to time it would have helped with the learning of the functions of the machine buttons as well as how to optimize the projections.

Seeing many echocardiographic images was a help. Something that both groups talked about was “visual and practice/theory” and the wish to see more images during the physics teaching, as well as when learning measurements, concepts and how to obtain projections.

#### Measurements and concepts

The students thought it was difficult to understand why, in what context and how certain measurements and concepts are used. Initially it was also hard to understand the meaning of the concepts. A suggestion was put forward to give students a list of the most frequently used measurements and concepts, including explanations of meaning but also why and when they are used. A manual for *how* to measure correctly would have helped. The students learned the concepts through the lectures and by studying the literature. YouTube films were also a support when it came to learning why and when certain measurements are used. The students also said that it would have helped to understand which measurements are used and when, if they had not only examined healthy persons but also those with heart disease. In addition, they desired more cases during lectures.

*F1S3: Well… it was the meaning of the concepts and what they are used for and why they are so important and… using it later when… well, when measuring.*

*F1S4: And how to measure them,*

*F1S3: Yes*

*F1S4: That was important too…*

*F1S3: That was the hard part to understand.*

In addition to the difficulty of learning measurements and concepts and making the connection to the projections was the concentration needed to obtain a projection and that it was so easy to lose a projection when the student had to think about measurements.

*F2S5: Yes, one was so concentrated on not losing the probe position which made it all even more difficult.*

## Discussion

Echocardiography is a clinically useful, important and globally used diagnostic examination of the heart’s anatomy and function. It is however notoriously difficult to learn. This study reveals several difficulties in the process of learning echocardiography – the projections, handling of the probe, why and how the measurements are executed and the practical application of the theory of ultrasound physics, to mention a few. Things that favour learning in echocardiography can be instant feedback, practical training (including “playing” with the ultrasound machine) and easily accessible pedagogical tools such as course literature, anatomical dummy as well as video lectures.

### Feedback

This study confirms the pedagogical benefits of feedback. The students wanted more instant feedback, especially at the beginning of the learning process, to receive confirmation about whether or not they were placing the probe correctly as this was one of the more challenging parts of the learning process. Hattie and Timperley [22] showed that feedback represents a central and effective part of influencing students’ learning. The students in this study suggested more teacher presence as a way of increasing instant feedback. Teachers, however, only have a limited amount of time for their teaching and increased teacher presence would result in increased costs. Furthermore, studies have shown that feedback from teachers, no matter how detailed it may be, in some cases do not lead to progression in learning. One of the reasons for this could be that students do not understand the feedback given to them [23]. Topping [24] showed that students often consider it easier to receive feedback from peers than from teachers. In this study the students highlighted the advantage of working together and helping each other at the ultrasound machine and acting as coaches for each other. The pedagogical advantage they experienced through this could be that they not only performed the examination but could also put forward their case about what they were doing and why.

One way to meet the students wish for more feedback could be to design a vLAB in such a way that it gives the student instant feedback [8, 25]. This might result in students being more open to feedback from the vLAB than from the teacher, in the same way as students are more open to feedback from their peers. To support them in their learning, the students in the current study wanted an independent assessor. By this they meant an assessor who was not involved in the course or in the final assessment of the students. A vLAB could perhaps act as this independent assessor. The feedback from an echocardiographic vLAB could include how the probe is placed and angled, where in the heart the ultrasound beam cuts and why, and also the quality of the measurements done. All of these points were problems that arose in the study. A vLAB provides the possibility to simultaneously show a 3D image of the heart and where the ultrasound beam cuts, while at the same time displaying a corresponding echocardiographic projection. That is something that may help in understanding projections. When relevant measurements on projections are to be done, a vLAB can indicate whether or not the measurement is correct, as well as present the user with fact boxes as to why and how the measurement is used. This feedback could be of value to the students who in the category “bridging the theory-practice gap” expressed difficulties in understanding why, when and how certain measurements are used. The vLAB cannot completely replace practical training but it can bridge the theory-practice gap. According to Dickson [26], interactive technologies can bridge the theory-practice gap within ultrasound teaching through the recurring feedback it can give students. A study showed that using a vLAB in the field of gynaecological ultrasound was good preparation for clinical practice [25].

The students’ desire for more feedback could also partly be satisfied by encouraging them to work three at a time at the ultrasound machine – one acting as “patient”, one operating the machine and one standing at the side. The one standing at the side can have access to pictures of the projections while asking the one doing the examination to find a certain projection and state what cardiac chambers are seen in the projections. The projections on the screen can be compared to the pictures of the projections and thus instant feedback can be given.

### Practical training and “playing”

In order to perform a clinically relevant, useful examination within echocardiography, one needs technical as well as practical skill. Handling the transducer is something often referred to as one of the more challenging parts of the examination [3]. The students were positive to being able to “play” with the machine and to the fact that there was no machine button that was “off limits”, hence it was a risk-free environment. Teaching methods can be split into two categories: guided and active/explorative training. In guided training the student is seen as a passive recipient of instructions while in active training the student is seen as an active participant in the learning process [27]. During practical training, active/exploratory learning demands minimal external guidance and, compared to guided training, it seems to promote metacognitive qualities in the student (ibid). Perhaps it was this active/exploratory training the students referred to when they said it was good to “play” with the machine. This “playing” can perhaps be developed further. One study showed that an effective way to promote learning is to deliberately integrate errors and mistakes in the active/exploratory training and that it was even more effective than “only” having active/exploratory training [28].

The students appreciated the possibility to practice on the machine in a risk-free environment where mistakes did not have serious consequences. Perhaps this can indicate that the students felt that the possibility of making mistakes was an important part of their learning. Since diagnostical mistakes can have serious consequences, the tradition in medical teaching has been to focus on how to avoid mistakes. However, it has been shown that diagnostical mistakes can be an important and positive part of medical learning and that the risk of making mistakes over a long time increases if they are not included in the learning process [29]. Instead of following the tradition in medical education of avoiding mistakes, students can be encouraged to make mistakes as a part of their learning. Dyre et al. [30] performed a study on medical students who had no previous experience of ultrasound and who were to learn transabdominal ultrasound by using a simulator. The students were divided into two groups – one group was encouraged to “play” with the machine and mistakes were regarded as positive, and one group was encouraged to follow the instructions from the simulator and make as few mistakes as possible. The results showed that after training with the simulator, the first group showed a better transfer of learning in a clinical environment.

By “playing” with the machine, making mistakes and actively learning echocardiography, the students develop their metacognitive ability as well as ability to solve problems. This can be used in future echocardiography teaching by consciously and actively encouraging students to make deliberate mistakes, find “new” projections and challenge one another to change the adjustments of the machine and then ask their fellow students to change them back etc. The limitation of the machine, however, is that it is fixed to a specific physical location and the students cannot access it whenever they wish. Also, it does not give any indication as to whether the transducer is placed correctly, whether the image quality is optimal or whether the measurements are acceptable, all of which a vLAB could be designed to do. A vLAB designed for giving feedback could therefore be combined with a function for “playing” with the machine. The feedback could be designed with the mindset that “mistakes are positive”. This kind of vLAB could therefore give the students more possibility to “play” and make mistakes but also receive instant and positive feedback in response to their mistakes, all in a stress-free, risk-free environment. Mistakes could help the students to identify their need of further learning within a certain area [29]. Mistakes made in a vLAB would not put patients at risk but rather prevent them from occurring in a clinical setting. Mistakes made within healthcare can be emotionally charged for those who make them. One further effect of teaching the mindset “mistakes are positive” is that the students not only learn from their actual mistakes but also learn how to handle their mistakes, leading to less stress if the mistakes are made in a clinical setting [31].

In this study the students expressed a desire to alternate their learning between theory and practice. Some students wished for practical training prior to theory in order to learn *how* and thereafter *why,* whereas some students wanted to know what they were doing before they held the transducer. One way to meet this wish is to use video lectures, coupled with free accessibility to the ultrasound machine. Our study reveals the benefits of video lectures and the students did suggest that the teachers could record some of the lectures. There are previous studies showing the appreciation of video lectures to traditional lectures when learning echocardiography. Tainter et al. [32] designed and evaluated a flipped classroom curriculum for teaching point-of-care echocardiography for residents rotating in the surgical ICU. Although the study was not designed to compare the traditional to the flipped classroom model, residents’ feedback expressed a preference for the flipped classroom model, compared to the traditional model. Previous studies have shown that watching video lectures prior to going into the laboratory increases the students’ knowledge of, confidence in and experience with certain laboratory techniques [33]. According to Makransky et al. [34], vLABs can work just as well as “face-to-face” lectures when preparing for a lab, suggesting a combination of virtual and physical labs within science education. Hence a vLAB perhaps can partly satisfy the students’ wish for more video lectures. However, one study assessed the impact of different teaching interventions when learning basic practical echocardiography skills by trying four different methods, one of which was video lectures. They found that even though the group learning through video lectures demonstrated assessment results similar to those of the other groups they still would have preferred a different teaching method altogether. One reason for this could be the fact that the other three groups in the study (peer-teaching, peer-teaching using Peyron’s four-step approach and team-based learning) all received feedback when learning the projection, unlike to the video group who only received technical support [35]. This may also endorse the findings of the benefits of feedback in this study.

### Limitations

This study has some limitations that may restrict or limit the transfer of findings. We acknowledge the limited number of participants, even though the two groups discussed similar themes, there might be a risk that not all possible themes have been covered. Also, only one educational programme from one university has been included in the study. Echocardiography is used by several professions and for different purposes making the curriculums, way of teaching and perhaps time spent learning different from one profession to another. However, this study focuses on the initial challenges when learning echocardiography- handling the probe and understanding the basics of ultrasound physics are the same for any profession learning echocardiography. Another possible limitation in this study was that the first author and the study participants had a previous examiner-student relationship outside the study. This may have led to the participants not feeling able to freely share their experiences from the course as they may have felt it could have a negative effect on their relationship with the main author. In order to avoid this, they were assured that the author would not allow the result of the study to affect their professional relationship and all students had passed the course and were informed of this prior to the interviews. On the other hand, the fact that the students were interviewed by someone they knew and who knew their echocardiographic background may well have made it easier and even more comfortable for them to share their experiences.

## Conclusions

This study shows the challenges when learning echocardiography and what might be helpful during the learning process. Main challenges when initially learning echocardiography are the projections and handling the probe as well as connecting ultrasound physics and measurements to practical application. Objective pedagogical suggestions for what might be helpful in the learning process such as; “playing” with the machine, immediate feedback, video lectures, the importance of swiftly alternating between practice and theory as well as the benefits of learning by mistakes in a risk-free environment are proposed. A vLAB within this field, designed to enhance that which is already helpful in the learning processes, could help to minimize or solve the difficulties in learning echocardiography.

### Practical implications

Based on the findings of this study, a vLAB within echocardiography could be designed to:

- Help students understand how to steer the transducer and how the beam should cut the heart in order to obtain a certain projection.
- Contain a virtual anatomical 3D dummy.
- Give indications as to whether the user’s measurements and projections are correct and of sufficiently high quality.
- Include fact boxes and questions concerning the physics and different measurements.
- Comprise various cases so that students would receive a greater understanding of pathology and what findings to expect.
- Help create a set-up where it is possible to swiftly alternate between practical experience and theory by using the vLAB together with hands-on practising with the ultrasound machine.

## Data Availability

The dataset used and/or analysed during the current study are available from the corresponding author on reasonable request. The dataset is in Swedish.

## Abbreviations

vLAB: Virtual laboratory
